# What influences the selection of contextual cues when starting a new routine behaviour? An exploratory study

**DOI:** 10.1186/s40359-020-0394-9

**Published:** 2020-03-30

**Authors:** Katarzyna Stawarz, Benjamin Gardner, Anna Cox, Ann Blandford

**Affiliations:** 1 Bristol Interaction Group, University of Bristol, Queen’s Building, University Walk, Bristol, BS8 1TR UK; 2 Department of Psychology, King’s College London, Denmark Hill, London, SE5 8AF UK; 3 UCL Interaction Centre, University College London, Gower Street, London, WC1E 6EA UK

**Keywords:** Contextual cues, Behaviour change, Habit formation, Digital interventions, Memory support

## Abstract

**Background:**

Contextual cues play an important role in facilitating behaviour change. They not only support memory but may also help to make the new behaviour automatic through the formation of new routines. However, previous research shows that when people start a new behaviour, they tend to select cues that lack effectiveness for prompting behaviour. Therefore, it is important to understand what influences cue selection, as this can help to identify acceptable cues, which in turn could inform future behaviour change interventions to help people select cues that best fit their context and so ensure continued repetition.

**Methods:**

We conducted a qualitative study to investigate what cues people select, how, and what influences their decisions. We recruited 39 participants and asked them to take vitamin C tablets daily for 3 weeks and later interviewed them about their experience. Quantitative habit strength and memory measures were taken for descriptive purposes.

**Results:**

Cue selection was primarily influenced by a desire to minimise effort, e.g. keeping related objects at hand or in a visible place; prior experience with similar behaviours (regardless of whether the cues used in the past were reliable or not); and beliefs about effective approaches. In addition, we found that suboptimal remembering strategies involved reliance on a single cue and loosely defined plans that do not specify cues. Moreover, for many participants, identifying optimal cues required trial and error, as people were rarely able to anticipate in advance what approach would work best for them.

**Conclusions:**

Future behaviour change interventions that rely on routine behaviours might fruitfully include the provision of educational information regarding what approaches are suboptimal (single factors, vaguely defined plans) and what is most likely to work (combining multiple clearly defined cues). They should also assess people’s existing beliefs about how to best remember specific behaviours as such beliefs can either enhance or inhibit the cues they select. Finally, interventions should account for the fact that early failures to remember are part of the process of developing a reliable remembering strategy and to be expected.

## Background

Many of the dominant causes of ill health and premature death can be linked back to everyday behaviours [1]. Simple actions such as eating more healthily, doing more physical activity, or adhering to medication, could improve health and wellbeing and extend lives [2, 3]. Effective behaviour change requires not only motivation, but also the skills and resources required to act [4]. Self-regulatory techniques, such as planning – i.e. identifying a situation in which to perform a specific behaviour – can help people to embed health behaviours into their daily routines [5, 6]. Action planning, which specifies what will be done and in what context, can aid not only initiation, but also maintenance of behaviour over time. Consistently performing a behaviour upon encountering a specific contextual cue reinforces cue-behaviour associations, which subsequently elicit the behaviour automatically and persistently (i.e. habitually [7]). As cue-behaviour associations strengthen, the control over the initiation of action is transferred from conscious intentional and memory processes to non-conscious processes, which are environmentally triggered [8, 9]. Action planning thus capitalises on salient features of everyday environments. Initially, environmental cues act as reminders, prompting people to consciously remember to act [10], but over time, as habit forms, they come to automatically trigger impulses that elicit behaviour outside conscious awareness [7, 11]. However, some cues may be more effective than others at prompting the behaviour [12].

### Effective contextual cues

How well cues and action plans support remembering depends on their components. For example, action plans (e.g. “I will take my pill after I eat my breakfast”) can refer to both the cue (“breakfast”) and the task (“take my pill”), refer only to the cue (e.g. “breakfast”) or refer only to the intention (e.g. “I will take the pill”). Although cues that simply remind that something needs to be done can be useful [13], plans that include both a cue and a task are the most effective at prompting the behaviour [14, 15] as they make clear what needs to be done and when [16]. Action plans that specify both a cue and a task, help to strengthen the mental association between the task and its cue [17], and increase the likelihood of the action being completed [17]. Existing routines may offer potent cues; in particular, combining tasks into a sequence may be an effective approach, such that one behaviour cues the next [18, 19], e.g. brushing teeth can serve as a cue to floss [20, 21]. Therefore, the boundaries between tasks within an existing sequence may be the best place to insert a new routine: the end of an action provides a strong cue and there is less competition from an established habit [22].

In addition, cues that become visible at the right time (as opposed to ones that are constantly visible) are more reliable as they attract attention at the most opportune moment for action [23, 24]. Similarly, distinctive cues that stand out (as opposed to nondistinctive ones that blend with the environment) are also easier to notice and thus are more effective at prompting memory [25]. Reminders are a good example of cues that can be made salient when needed. However, while some studies demonstrate the effectiveness of reminders in supporting prospective memory (e.g. [26]), other research shows no benefits (e.g. [14, 15, 23]). In addition, people who expect to be reminded score worse in prospective memory tests [27], as they invest less mental effort in trying to remember and are more likely to forget. The effectiveness and salience of reminders decreases with time [11], which makes them less reliable for long-term regular tasks as they can become too familiar and people can learn to ignore them [11]. Moreover, the use of reminders can inhibit the process of habit formation [28] by leading to the development of a dependency on the presence of reminder, meaning that the routine may be discontinued when they are gone [29].

Despite evidence on the properties of effective cues, people tend to select suboptimal ones. For example, in a study on healthy eating habits, Gardner et al. [30] discovered that two-thirds of participants specified suboptimal cues when formulating action plans, e.g. did not link the behaviour with a specific routine (e.g. “a cup of milk every day”), and failed to specify exactly what needed to be done and when (e.g. “extra water during the day”). When they did specify cues, they often tried to pursue multiple behaviours in response to multiple cues (e.g. “juice with breakfast, water with lunch and dinner and milk at bedtime”), which can prevent the development of cue-behaviour associations. The authors did not explicitly explore the reasons for selecting these cues, although post-hoc discussions suggested participants lacked insight into which cues would be effective in this context.

## The present study

Understanding how and why people use environmental cues in their everyday lives, and to what effect, may help efforts to develop effective interventions for using environmental cues to support behaviour change, by highlighting which cues are most acceptable to people. We conducted a 3-week qualitative study with semi-structured interviews to explore people’s everyday behaviours and gain more in-depth understanding of how they select cues and what influences their cue decisions. As an example of a new daily task, we promoted taking a daily vitamin C tablet, a behaviour that has previously been used in studies exploring how people remember new behaviours [31] or form habits [32].

### Method

#### Participants

Through social media and leaflets distributed on a university campus in the United Kingdom, we recruited 39 participants who were willing to start taking vitamin C supplements. As taking tablets and other medications can be a habitual behaviour [33], it is possible that strategies developed early could influence future strategies. Therefore, we decided to focus on younger people who may not have well-established long-term medication routines as only 19% of young adults aged 16–24 take prescribed medication vs 90% of those aged over 75 years old [34]. However, for comparison purposes and to enable a deeper understanding of factors that influence cue selection, we did not exclude participants who might have already developed routines for other medications.

#### Materials

Each participant received a box of 30 × 200 mg vitamin C tablets available over the counter in a high street pharmacy. We used these specific tablets to minimise potential risks, as taking less than 1000 mg of vitamin C supplements daily is unlikely to cause any harm [35]. The tablets were chewable and did not require access to water, thus making the participants’ task as simple as possible.

Participants were recruited through a website that included the information about the study. After reading this information and consenting to participate, participants were redirected to the recruitment form that collected background information. It included two standardised questionnaires: the Prospective and Retrospective Memory Questionnaire (PRMQ) [36] and the 4-item Self-Report Behavioural Automaticity Index (SRBAI) [37]. PRMQ was used to assess participants’ memory and identify those whose memory was either ‘below’ or ‘above’ average, i.e. whose scores fell within two standard deviations from the median scores of the control group from [36]. SRBAI was used to quantify the automaticity of medication-taking behaviour of participants who had already been taking daily medications. SRBAI captures automaticity through four statements that follow a stem: (“Behaviour X is something …” ) “I do automatically”, “I do without having to consciously remember”, “I do without thinking”, and “I start doing before I realise I’m doing it”. It is an automaticity subscale of the Self-Report Habit Index (SRHI) [38], which is a validated questionnaire that has been used in over a hundred of studies [39]. It includes 12 statements that, apart from automaticity of behaviour, help to also capture frequency (e.g. “… I do frequently”) and sense of identity (e.g. “… that’s typically ‘me’”). As SRHI touches on these various aspects of habitual behaviours, we used it at the end of the final interview to further guide the discussion. Using SRHI at this point also allowed us to extract final SRBAI scores to compare with the scores collected at the beginning of the study in order to assess whether our participants had developed a habit of taking the vitamins.

#### Procedure

We set up a recruitment website where participants were able to read the information sheet and sign up for the study and indicate their consent. Those who consented were then asked to complete the PRMQ and SRBAI questionnaires and to provide their email address to allow researchers to get in touch with them. After receiving participants’ contact details, we emailed them a link to an online booking page with available interview slots to let them choose the most appropriate time for the first interview.

Each participant was interviewed twice. The first interview explored everyday remembering practices, including remembering healthy behaviours and strategies to prevent forgetfulness, as well as participants’ experiences with remembering medications, causes and frequency of forgetting, and plans for remembering vitamin C tablets during the study (see Additional file 1 for the interview guide). At the end of this interview participants received a box of vitamin C tablets and were instructed to take one every day until the next interview. They were asked not to take any unusual steps to support their memory during the study, e.g. not to use reminders if they normally do not use them for medications. A date of the follow-up interview, around 21 days after the first interview, was also agreed.

**Fig. 1:**
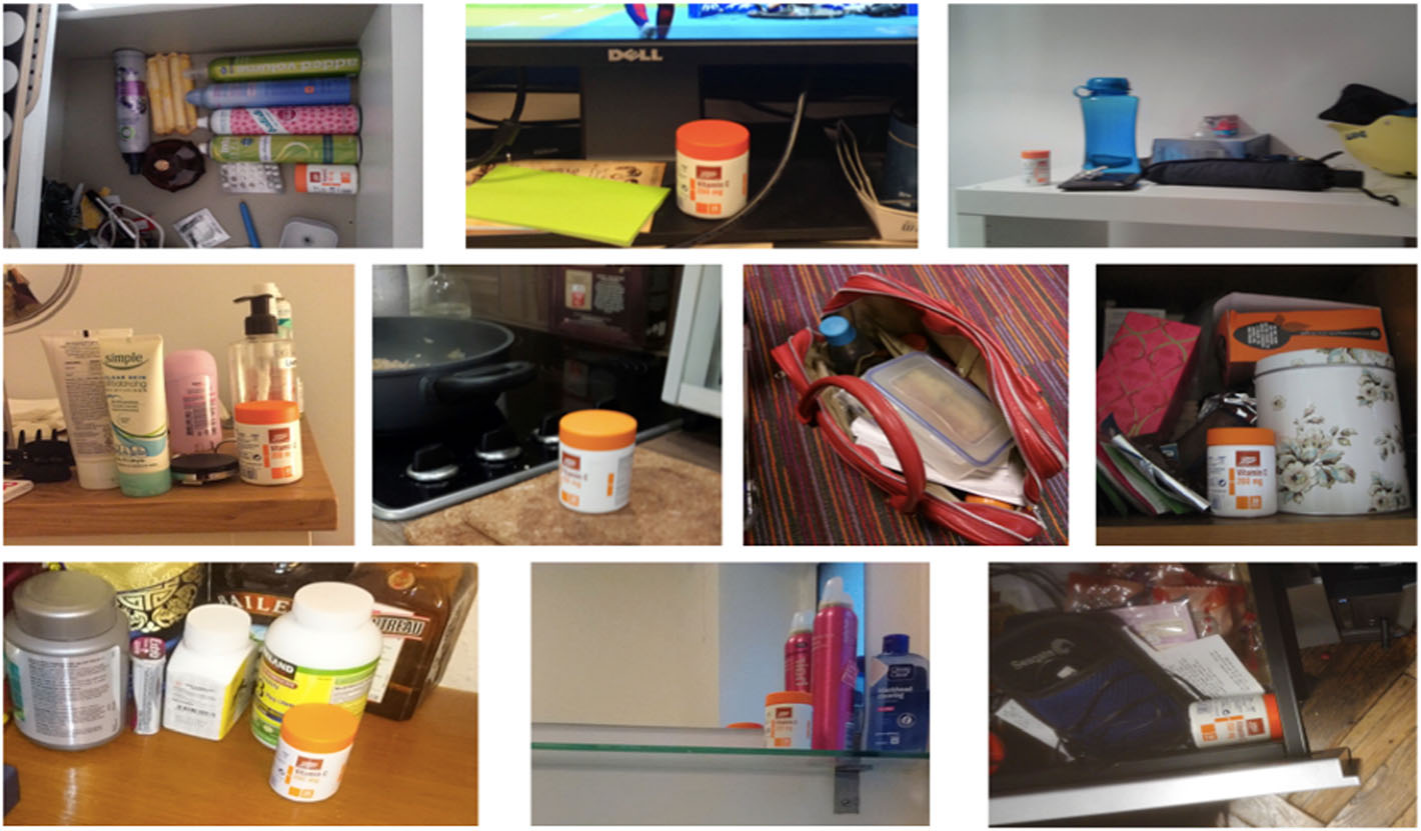
Examples of locations and objects that helped participants remember their vitamins

About a week before the second interview, we asked participants to send us a photo showing where they kept the vitamins to aid the discussion during the second interview (example photos are shown in Fig. 1). The second interview focused on experiences of attempting to take vitamin C tablets during the intervening period. It explored remembering strategies, rationale for selecting specific cues, ease of remembering, and reasons for forgetting (see Additional file 1 for the interview guide). The photos sent by participants only served as interview prompts and were not used in the analysis. At the end of the interview participants were also asked to fill in the SRHI questionnaire to assess habit strength for vitamin-taking and to explain their responses, which allowed for more in-depth reflection regarding their cue choices. Each participant who attended the second interview received a £15 (US$20) shopping voucher.

#### Analysis

Both interviews were audio-recorded and transcribed verbatim. Initial notes were taken during the transcription and the first reading. Thematic analysis [40] was used to analyse the transcripts, following both top-down and bottom-up approaches. Results of earlier studies that explored strategies for remembering medications (e.g. [41–44]) informed the coding frame that was used in the top-down analysis. Upon the second reading, parts of the transcripts related to general remembering strategies and forgetfulness (first interview) and remembering vitamin C (second interview) were coded at a sentence level, and general impressions and thoughts were noted. Pre-defined code categories included: everyday remembering strategies, reasons for forgetting, information related to current medications, information related to past medications, strategies for remembering vitamins during the study, and information about forgetting them. In parallel, a bottom-up approach helped to identify new trends and issues, including the role of visual cues, problems with automatic behaviour, and the differences between initial plans for remembering vitamins and the actual strategies that were used. After the coding was finished, similar codes were merged or grouped together under the same label, and additional codes were defined. Coding was done by the first author and regularly discussed with co-authors throughout the process. NVivo 10 for Mac [45] was used to code, annotate, and analyse the transcripts.

We also calculated participants’ adherence based on their reports of forgetting. This was mainly based on self-reports, although 10 participants brought their vitamin C boxes and counted the remaining tablets during the final interview, and two counted them at home and emailed the exact number to the research team.

### Results

#### Participant characteristics

Of the 39 participants who started the study, 38 completed it and their details are summarised in Table 1. One participant did not attend the second interview and so their data was excluded. Participants who completed the study were 18–43 years old (mean = 23 years, SD = 3.7); 20 were male (51%). Half were undergraduate students (20 participants). Six participants reported currently taking long-term medications, 17 had taken long-term medications in the past, six only had experience with short-term regimens, another six had not taken any medications since moving out from their parents’ house, and three reported no relevant experience at all. See Table 1 for details.

**Table 1:** Remembering strategies and incidents of forgetting reported by participants who completed the study (*N* = 38)

#	Age / gender	Previous medication-taking experience	Cues used during the study (location and objects; time or routine)	No. of missed tablets
P1	19/F	Childhood; fish oil, herbal medicines	Desk, with toiletries; morning routine	3–4
P2	20/M	Short-term; antibiotics	Desk; morning or afternoon	1
P3	20/M	No experience	Many changes to find the right approach	1
P4	29/M	Long-term; supplements, post-surgery	With wallet, keys, bike helmet; after coffee	0
P5	20/F	Long-term; contraceptive pill	On a make-up table; morning routine	1
P6	21/M	Childhood; vitamin C, supplements	Desk; whenever remembered	10+
P7	19/M	Childhood; vitamin C	Next to tea cup; with evening meal	1–2
P8	27/M	Long-term; vitamin C	Desk, with keys; morning	2
P9	22/F	Currently taking; homeopathic pills	Desk; before leaving in the morning	1–2
P10	19/F	Currently taking; contraceptive pill^a^	In a bag, with contraceptive pills; reminder	0
P11	21/M	Short-term; antibiotics	By the bed, with keys and wallet	0
P12	20/M	Childhood; herbal medicines	First on a chest of drawers, later inside	10+
P13	29/F	Long-term; herbal medicines	In a bag; times varied	2–3
P14	26/M	Long-term; malaria pills, multivitamins	By the bed; when getting dressed	2–3
P15	25/M	Long-term; weight loss pills	Desk; in the morning	0
P16	22/M	Short-term; antibiotics, painkillers	Bathroom; after brushing teeth	0
P17	20/F	Childhood; herbal medicines	Desk; after breakfast	0
P18	18/F	Short-term; cold and flu medicine	Tested a different approach each week	4–5
P19	24/M	No experience	Bathroom; after brushing teeth	7
P20	24/M	Long-term; herbal medicines	Kitchen, next to stove; with breakfast	1–3
P21	23/M	Childhood; herbal medicines	Desk, next to laptop	1–2
P22	22/F	Long-term; supplements	First in a bag, then on the desk	2
P23	31/F	Long-term; fish oil, contraceptive pill	Tea cupboard; with morning tea	5–6
P24	34/F	Currently taking; thyroid medications^a^	Jacket’s pocket; when needed a break	5
P25	25/M	Long-term; supplements	Office desk’s drawer; at 11 am	1
P26	24/F	Long-term; supplements	Shelf by the bed; morning	0
P27	22/F	Childhood; multivitamins, supplements	Backpack; with laptop charger	1
P28	20/F	Currently taking; contraceptive pill^a^	Drawer, with contraceptive pills; morning	1–2^b^
P29	19/M	Long-term; vitamin C, hay fever pills	Kitchen, medicine drawer; breakfast	0^b^
P30	26/F	Long-term; malaria pills, contraception	Backpack; whenever remembered	1
P31	19/M	No experience	After dinner, before brushing teeth	0
P32	24/M	Short-term; cold and flu medicines	Office desk; whenever remembered	1
P33	28/F	Long-term; supplements, contraception	Bag; on a bus to work	1^b^
P34	21/F	Currently taking; contraceptive pill	With contraceptive pill, after alarm clock	1
P35	19/M	Long-term; vitamin C	Next to bed; whenever remembered	10 + ^b^
P36	21/M	Short-term; antibiotics	Next to phone; before leaving	2
P38	20/F	Currently taking; herbal medicines^a^	After breakfast; parents prepared	4–5
P39	24/F	Long-term; contraceptive pill	Office desk; after coming to work	4

Participants whose existing medication regimens were automatic (based on their SRBAI scores) are marked with ^a^. Those with below average memory (based on their PRMQ scores) compared to the control group from [36] are marked with ^b^. Number of tablets missed is based on self-reports or tablet counts. P37 did not attend the second interview and their data was excluded

Participants’ reported adherence varied from 35 to 100%, with most (*N* = 34, 89%) reporting adherence over 71% – an equivalent of remembering every weekday. PRMQ scores showed that most participants had an average memory, i.e. their scores fell within two standard deviations from the median scores of the PRMQ control group from [36]. Only four participants’ scores fell outside that range, indicating that their memory was below average. However, during the study there were no differences in rates of forgetting or remembering strategies between them and the rest of the participants (see Table 1). The SRBAI scores extracted from the SRHI questionnaire completed in the final interview indicated that 11 participants had started to develop automaticity (SRBAI scores of 14–16 out of 28), but the behaviour did not become fully automatic. Table 2 summarises different combinations of cues (which are reported in detail in Table 1) in relation to SRBAI scores and self-reported adherence.

**Table 2:** Different combinations of cues with relation to SRBAI scores and self-reported adherence

Cue type	N	SRBAI		Self-reported adherence	
		Mean	SD	Mean	SD
Routine only	1	8	–	80%	–
Location only	11	12	2.6	87%	14%
Routine + location	11	12	3.0	90%	12%
Location + object	3	10	2.9	75%	35%
Routine + location + object	12	12	3.6	92%	8.3%
Reminder	1	9	–	100%	–

SRBAI scores range from 4 to 28; higher scores indicated stronger habit and automaticity

#### Remembering strategies

In line with prior research, participants reported relying on contextual cues: routine events, locations, and objects (see Table 2). Figure 1 illustrates some of their strategies. The most common strategy was the use of multiple cues. For example, P4 kept vitamins with his keys and wallet, and would take one after his morning coffee when getting ready to leave the house, while P16 kept vitamins in the bathroom and took one every morning after brushing his teeth. Only one participant (P10) reported using reminders as the main cue as she had already been using a reminder for the contraceptive pill and decided to take vitamins together with it (and therefore in practice used a combination of contextual cues and a reminder). In addition, another participant (P18) reported that she decided to experiment with using a reminder in the final week of the study to test whether she would like it, reportedly having not previously used one for medications; however, for the rest of the study she used different contextual cues (meals, other pills, etc).

#### Identified themes

We identified four themes describing the factors relevant to cue selection. When selecting cues, participants aimed to minimise effort required to remember the new behaviour and relied on prior experience and beliefs to guide their choices. The analysis also highlighted the need for trial and error when developing a new remembering strategy and allowed us to identify the characteristics of suboptimal strategies. All are described below with representative quotations. Figure 1 illustrates some of the strategies discussed during the interviews.

##### Theme 1: minimising effort as the primary remembering strategy.

Many participants selected strategies that would make remembering as easy as possible. This was often limited to simply making the vitamins visible, as participants believed that this would be sufficient to help them remember to take the tablets on a regular basis:

*“When I see it, I take it – that’s the plan.”* – P15, 2^nd^ interview

*“I think I made a decision to take one by my eye, but not by my mind.”* – P32, 2^nd^ interview

However, while relying on visual cues made remembering easier by making the cue very salient and permanently present in the environment, this approach had its limitations:

*“As I saw it, I would take it automatically. I didn’t think about it, because I was thinking about something. And then yeah, sometimes I would be looking at it and thinking ‘I will take it’ and then … I would remember that actually I* [already] *took it today.”* – P21, 2^nd^ interview

Participants’ experience suggested that using the vitamin box as a visual cue was more effective when it was kept next to specific objects they used daily.

*“I put it next to my keys, the kind of things I have to take every day, so it’s very easy to see it and to take it.”* – P4, 2^nd^ interview

The prioritisation of effort minimisation was also reflected in using a combination of existing routine actions and a strategic location. Participants felt that they had to make minimal adjustments to their existing routines, e.g. add only one extra step.

*“It’s next to my toothbrush* [ … ] *I brush my teeth and then I put my toothbrush away and then I take my tablet. And because it was like right next to it, and I’m focusing on the toothbrush, and that’s where it is, then it would be... I’d remember it more, because it’s like the next step kind of thing. So I tried to incorporate it into my daily routine with the toothbrush. That went quite well.”* – P18, 2^nd^ interview

Reducing effort also meant that participants selected or considered selecting cues that would provide additional safeguards that would also make it easy to take missed doses in case they forgot.

*“I’d probably keep it in my bag and then at least if I’ve forgotten during the day, I’ve got them with me.”* – P11, 1^st^ interview

People who already had been taking medications regularly decided to add vitamin C tablets to their existing regimen. As such, they felt it was the easiest way to remember the vitamins: with a remembering strategy already in place, it did not require any extra effort.

*“I made it easier because they were both* [the contraceptive pill and vitamin C] *at the same time, so I can take two pills and I’m good. For the first few days I had to definitely remind myself to take* [vitamins]*, but closer to the end I was like ‘okay, do I have two? right.’ ”* – P10, 2^nd^ interview

##### Theme 2: prior experience and beliefs influence remembering strategies.

Many participants reported using strategies that they felt were proven and tested – that is how they remembered things in general or how they used to remember medications in the past.

*“I was ill a couple of months ago and I had a cold, and I took some tablets. I kept them on my desk and on my bedside table, so I remembered to take them every 4 hours. So that’s the way I tend to remember medicine.”* – P18, 1^st^ interview

However, the existing tasks used as cues sometimes did not prompt the action, as they were part of very specific routines that participants did not always follow exactly in the same way. Some participants were aware of this limitation from the start, but the possibility of missing a few tablets did not bother them:

*“If I have to take something every day, I always put it in the coffee and tea cupboard.* [ … ] That’s the best place in which I remember. I mean, there’s always one or two mornings when I don’t take coffee or tea, but … so I will forget *laughs*” – P23, 1^st^ interview

Others believed that their approach would work, even if it had not in the past. For example, one participant reported previously taking vitamin C tablets and almost never being able to remember them, despite their placement in a visible spot on his desk. Nevertheless, this is exactly how he tried to remember vitamins during the study:

*“I think if you give me* [the vitamins]*, I will feel that I would be like kind of obliged. Because I committed myself to that. So I think the motivation is actually to fulfil what I committed myself to, not my health.”* – P35, 1^st^ interview

In the end, P35 reported missing tablets on more than 10 occasions. Such beliefs about motivation and how people remember things, vitamins in particular, were another factor that influenced the selection of cues. For example, some participants believed that vitamins had to be taken at a specific time of day (in the morning, with meals, before sleep, etc.) and as such they had no other choice but to take the vitamins at this time, even if it was not optimal or did not match their routine.

*“They say that the vitamin is the best before breakfast. So I’ll probably have it then.”* – P15, 1^st^ interview

*“I think vitamin C is a morning thing, because you associate it with orange juice and that sort of thing* [ … ] *something about the colour makes it about the morning.”* – P11, 1^st^ interview

*“Someone said that it is not good to take* [vitamins] *after the caffeine* [ … ] *and also at night our body is sleeping more, so it is great because nothing else is going inside, so taking the tablet at night* [is] *more useful.”* – P13, 1^st^ interview

Similarly, the beliefs about the best placement of medications influenced participants’ choices, even though it could potentially hinder remembering, e.g. when their beliefs dictated that medications had to be hidden from sight.

*“I normally put* [pills] *beside my bed* [ … ] *I guess that’s a routine for people on medicine. I think they always put it there.”* – P11, 1^st^ interview

*“I don’t think it’s a lucky thing to keep medicine in a visible place.”* – P32, 1^st^ interview

##### Theme 3: trial and error as an important part of identifying the right cues.

The interviews showed that participants had a difficulty anticipating what strategies would work form them. During the first interview, 37 participants discussed their plans for remembering vitamins; however, only 27 actually followed that plan for at least a day, including only two participants who used the same cues throughout the whole study (P34 kept vitamins with the contraceptive pill and P38 with ginseng tablets). Others either started with a completely new approach or adapted their initial plan to make it work.

*“It would probably be with whatever meal I'm eating at home. So it would be either breakfast or dinner, because I'm used to taking medications around these times.”* – P1, 1^st^ interview

*“I keep it with everything that I use in the morning* [ … ] *At first it was at the back of the* [make up] *table, at the back of the shelf, but then I moved it to the front.”* – P1, 2^nd^ interview

*“Well, I'll probably just put them in my bathroom.”* – P33, 1^st^ interview

*“So I kept* [vitamins] *in my bag, tried that out and I would take* [them] *on a bus.”* – P33, 2^nd^ interview

For some, the change happened naturally, others tried multiple options, while for a few participants finding the right cues was a long process of trial and error. Nevertheless, sooner or later they all found a satisfactory approach.

*“It was on my desk [on the first] day. But* [...] *when I went to brush my teeth I took it* [to the bathroom]*, and then it remained there.”* – P16, 2^nd^ interview

*“The first place I had it was in the kitchen* [but] *if I didn’t have breakfast, I wouldn’t have it, I wouldn’t go there and I would forget about it* [ … ] *Then I put it next to my work desk.* [ … ] *And then, eventually, I moved it to my bed* [ … ] *Because sometimes I’m thinking, ‘did I take it? or did I not take it?’ And so I* [ … ] *made a choice* [that] *I will just wake up and take it straight away. So that’s why it finally got on top of my drawer, but that took me 2 weeks, maybe a bit less, to move it, to place it actually there.”* – P3, 2^nd^ interview

##### Theme 4: characteristics of suboptimal cues.

The data highlighted two intertwined factors that reduced the effectiveness of remembering strategies: reliance on a single cue and vaguely specified plans. Using a single cue, regardless of whether it was a location, an object or a routine, made the remembering strategy too vulnerable: if the participant did not encounter that cue, they would forget. For example, one participant’s cue was wearing a specific jacket and hearing the sound of the vitamins in the pocket, but this cue relied on external factors: weather (what to wear?) and leaving the house in the first place.

*“I kept the* [vitamin] *box in my jacket, so I could hear the click click click* [when I walked]*, so I always thought about it.* [ … ] *I’ve changed my jacket, so the box got stuck in the other jacket, so I forgot once or twice.”* – P24, 2^nd^ interview

Similarly, another participant kept the box of vitamins in her backpack together with a laptop charger. Her plan was to take a vitamin when she takes the charger out; this was her only cue as she used the charger at different times each day. However, when she did not need to charge her laptop, she missed the vitamins:

*“I think I forgot to take one* [tablet]*, because I think that day I was so busy I didn’t use my charger.”* – P27, 2^nd^ interview

Such reliance on ill-specified cues was often ineffective. All participants who reported missing tablets on more than seven occasions (a third of all tablets they were supposed to take) had no structured remembering strategy and their approach could be summarised as “I’ll take it when I see it”. This lack of structure not only made remembering more difficult, but also sometimes caused uncertainty around whether the tablet had already been taken that day.

*“I would walk around my room and it would catch my eye and I would be like ‘did I take it or not?’ ”* – P1, 2^nd^ interview

*“I think a couple of days I probably took one and later was like ‘did I take it once a day?’, so I guess some days I took two instead of missing out.”* – P14, 2^nd^ interview

On the other hand, all participants who reported not missing a single tablet reported relying on multiple cues. They often reported that taking vitamins had become part of their routine and that clearly defined cues helped them remember the vitamins when their environment changed. However, while combinations of multiple cues were the most effective, these cues had to be unique – otherwise encountering the same combination at a later point during the day caused doubts. Similarly, getting too used to taking the vitamins sometimes caused difficulties with remembering whether the tablet had already been taken.

*“It was a couple of times where I looked at the table, “did I take it this morning?”, but I couldn’t remember, so I sort of left. And just thought, that it’s confusing.”* – P11, 2^nd^ interview

## Discussion

This study sought to understand what cues people select and what influences their choices. We identified four themes relating to influences on cue selection (aiming to minimise effort, prior experience, and beliefs), including factors that reportedly reduced the effectiveness of remembering strategies: reliance on a single cue and loosely defined strategies, which suggest that combinations of clearly defined cues are the most useful. Moreover, many participants seemed not to anticipate which cues would work, and instead used trial and error to find the most appropriate cue.

In general, participants did not give much thought to how they were going to remember the vitamins and often assumed that as long as they could see the vitamin box, they would remember to take the tablets. However, while visual cues can be effective [25], it is likely due to their salience. Visual cues that are continually visible lose their salience and, as a result, their effectiveness [23]. Indeed, participants who encountered their cues on multiple occasions throughout the day were often unable to remember whether they had already taken the tablet that day. Moreover, when one has to regularly repeat a task, it can be easy to confuse thinking about it with the memory of completing it [46]. Participants reported this type of error, which could be attributed to the regular exposure to the vitamin box that served as a visual cue. This also highlights participants’ inability to define specific cues that would help them take vitamins every day and remember whether a tablet had already been taken. This issue could have been addressed by using a reminder, but only two participants reported using them.

Participants sometimes wanted to follow their past strategies, regardless of whether they had worked or not, perhaps because they lacked capability to identify ‘better’ cues. This might alternatively have been a result of optimism bias, such that, despite previous failures, people remained confident that they would remember to act in response to these cues in the future. The difficulty in selecting optimal cues was further reflected in plans for remembering the vitamins reported in the first interview: very few participants had a specific plan in mind, and in most cases it was swiftly abandoned or modified. This lack of structure made the remembering strategies vulnerable to changes in participants’ daily life and they missed the tablets when they did not visit a specific location, left the home earlier or, in one case, did not wear specific clothes. This is in line with existing research that shows that forming general goals is less effective at ensuring the task gets done than forming more specific if-then plans [47, 48], which was also shown to be the case for remembering vitamin supplements [31]. In addition, if a plan was based on a single cue (regardless of whether it was linked to a location, an object, or a routine), it introduced dependency: if the cue was not encountered, the participant missed the tablet, which sometimes prompted changes to their approach and the search for better cues. However, as in previous research [30], participants did not seem to understand what constitutes a good cue and often had to change their plan to ensure vitamins would fit into their daily routine. Since people may not recognise the cues that prompt their behaviour [49], it is also possible that they may not fully understand their routines. As the cues people plan to use or choose initially are seldom the ones that help them remember in the long term, some degree of individual trial and error experimentation is a necessary step in the formation of good remembering strategies. This experimentation could help to develop a better understanding of which cues work for each individual and how they fit into their everyday life.

Overall, our results suggest that behaviour change and habit formation interventions should provide information about the importance of contextual cues and examples of ‘good’ cues to help people define their own; that is, cues should be unique, related to the behaviour and ideally part of a cluster of multiple combined cues. Given that our participants aimed to put as little effort as possible into remembering the new behaviour, interventions might include examples of specific routines a person can adopt, based on prior studies that assessed the effectiveness of different approaches. They could also include examples of what people usually do; this would not only illustrate cues that are commonly acceptable to people, but also provide inspiration and reinforce the beliefs regarding the expected behaviour. However, any such intervention should make clear that finding the right cues can take time and may require trial and error; occasional forgetting is to be expected. Moreover, as the conduciveness of cues to action is dependent on individuals and contexts, the intervention should only point people towards ‘better’ cues if the ones they have selected are suboptimal; if their cues help them engage with the behaviour, there is no need to promote the use of certain cues.

Limitations of our study must be acknowledged. While our findings shed light on characteristics of ‘good’ cues, it is difficult to identify the mechanisms by which participants’ chosen cues may have worked, e.g. they may have directly cued representations of behaviour, so acting via habit pathways [9, 50], or they may have reminded people of their goals, which in turn prompted action [51, 52]. Given the nature of the research question and the interest in people’s everyday behaviour, our data was based on self-reports, and people may not always be honest or accurate when talking about adherence [53, 54]. However, patients perceive forgetfulness as more socially acceptable than admitting intentional non-adherence [55, 56] and so when they do describe their non-adherence and reasons for forgetting, as our participants did, their reports are often accurate [57]. Moreover, we were more interested in understanding how people select their cues, rather than actual adherence rates, and the photos sent by participants provided a reliable record of their approach. Nonetheless, if people lack accuracy in recalling adherence, they may also lack accuracy in recalling cues or their impact; therefore, future work might seek to use objective measures of adherence and cueing (e.g. via sensors).

We focused on a younger population, mostly students. However, our findings may be generalisable as prior research shows that people use the same types of cues regardless of their age or regimen [44], which suggests that the motivations for selecting cues may be similar as well. Moreover, the cues our participants selected reflected those reported in previous studies conducted with other populations [41–44].

## Conclusion

Our study explored how people who start a routine behaviour select cues that are meant to help them adhere to it and what factors influence this selection. Our work confirms and expands existing research. The results show that the desire to minimise effort, previous experience, and beliefs influence cue selection; and that people cannot always anticipate whether the cues they would like to use will turn out to be effective. Moreover, given that several participants used the same approach they had used in the past, this suggests that these early experiences may determine which cues will be used in the future in a similar context. Therefore, it is likely that people use cues they have learned over the course of their lives, which makes it even more important to understand how they select them in the first place and to educate them on effective remembering strategies. Future behaviour change interventions should take these factors into account and provide information on characteristics of both good and suboptimal cues. They should also allow for experimentation to enable people to find the cues that best fit into their daily routines.

## Supplementary information

**Additional file 1.** Semi-structured interview guides. Interview guides used in the initial and final interviews.

## Data Availability

The datasets used and analysed during the study are available from the corresponding author on reasonable request.

## Abbreviations

PRMQ: Prospective and Retrospective Memory Questionnaire
SRBAI: Self-Report Behavioural Automaticity Index
SRHI: Self-Report Habit Index
