# Supplementary material for: What influences the selection of contextual cues when starting a new routine behaviour? An exploratory study
Source: BMC Psychol. 2020 Mar 30;8:29. doi: 10.1186/s40359-020-0394-9 (PMC7106637; doi:10.1186/s40359-020-0394-9)
Supplement: Supplementary file 1 — Additional file 1. Semi-structured interview guides. Interview guides used in the initial and final interviews. [file 40359_2020_394_MOESM1_ESM.pdf]

Semi-structured interview guides

Initial interview

- What do you study? Do you have a fixed schedule? (i.e. wake up at the same time very day?) How do you remember everyday tasks?
- What healthy behaviours do you engage in on a regular basis? e.g. exercises, walking, drinking water, eating fruit and veg, flossing)
  - How often? How do you remember?
  - Do you forget? If so/if not, why?
- Do you take any medications? (or used to take in the past)
  - How often?
  - Where do you keep them? – What helps you remember?
- Do you ever forget your medications? If so/if not, why?
- When starting a new regular task, how do you decide what is going to help you remember?
- How do you think you're going to remember the vitamins?
- Why would you like to take the vitamins?

Post-study interview

- How was it? How did you remember?
  - Where did you keep the vitamins? Did you keep them there from the start?
  - What time did you take them? Did you try other times?
  - What helped you remember?
  - Were there any issues?
  - Talk about the photo
- Was there a difference in how you remembered it during the first week and now?
- Was there a difference between weekdays and weekends?
- How many times did you forget? Why?
- Have you ever taken more than one in a day? Why?
- What do you think might have helped you to remember better?
- SRHI questionnaire – explain your answers
- Are you planning to continue taking the vitamins?
- Has anything changed in the past 3 weeks in terms of healthy behaviours?
- What was your motivation to participate in the study?
